# Functional Expression of Transient Receptor Potential and Piezo1 Channels in Cultured Interstitial Cells of Human-Bladder Lamina Propria

**DOI:** 10.3389/fphys.2021.762847

**Published:** 2022-01-06

**Authors:** MengMeng Zhao, Zhenghao Chen, Lei Liu, Ning Ding, Jiliang Wen, Jiaxin Liu, WenZhen Wang, Nan Ge, Shulu Zu, Wei Song, Guoqing Chen, Xiulin Zhang

**Affiliations:** ^1^Department of Urology, The Second Hospital, Cheeloo College of Medicine, Shandong University, Jinan, China; ^2^Department of Urology, Friendship Hospital, Capital Medical University, Beijing, China; ^3^Department of Urology, Shandong Provincial Hospital Affiliated to Shandong First Medical University, Jinan, China; ^4^Department of Urology, China Rehabilitation Research Center, School of Rehabilitation, Capital Medical University, Beijing, China

**Keywords:** bladder interstitial cells, Ca^2+^ imaging, lamina propria, Piezo channel, TRP channel

## Abstract

The interstitial cells in bladder lamina propria (LP-ICs) are believed to be involved in sensing/afferent signaling in bladder mucosa. Transient receptor potential (TRP) cation channels act as mechano- or chemo-sensors and may underlie some of the sensing function of bladder LP-ICs. We aimed to investigate the molecular and functional expression of TRP channels implicated in bladder sensory function and Piezo1/Piezo2 channels in cultured LP-ICs of the human bladder. Bladder tissues were obtained from patients undergoing cystectomy. LP-ICs were isolated and cultured, and used for real-time reverse transcription-quantitative polymerase chain reaction, immunocytochemistry, and calcium-imaging experiments. At the mRNA level, TRPA1, TRPV2, and Piezo1 were expressed most abundantly. Immunocytochemical staining showed protein expression of TRPA1, TRPV1, TRPV2, TRPV4, TRPM8, as well as Piezo1 and Piezo2. Calcium imaging using channel agonists/antagonists provided evidence for functional expression of TRPA1, TRPV2, TRPV4, Piezo1, but not of TRPV1 or TRPM8. Activation of these channels with their agonist resulted in release of adenosine triphosphate (ATP) from LP-ICs. Inhibition of TRPV2, TRPV4 and Piezo1 blocked the stretch induced intracellular Ca^2+^ increase. Whereas inhibition of TRPA1 blocked H_2_O_2_ evoked response in LP-ICs. Our results suggest LP-ICs of the bladder can perceive stretch or chemical stimuli *via* activation of TRPV2, TRPV4, Piezo1 and TRPA1 channels. LP-ICs may work together with urothelial cells for perception and transduction of mechanical or chemical signals in human-bladder mucosa.

## Introduction

Interstitial cells (ICs) in the bladder have attracted much research attention in recent years (Andersson and Mccloskey, 2014; Koh et al., 2018). Based on their location, two populations of ICs in the bladder have been characterized: LP-ICs, which are between the urothelium and detrusor and ICs in the detrusor (detrusor-ICs) (Andersson and Mccloskey, 2014; Gevaert et al., 2014). LP-ICs can be sub-grouped further into ICs in the upper lamina propria (ULP-ICs, which are immediately beneath the urothelium) or sub-urothelial ICs and ICs in the deep lamina propria (DLP-ICs, which lie between the ULP and detrusor) (Gevaert et al., 2014).

Several molecular markers are used to characterize bladder ICs: the broad mesenchymal marker vimentin (Vim) (Davidson and Mccloskey, 2005; Gevaert et al., 2014), the myogenic differentiation marker alpha-smooth muscle actin (Monaghan et al., 2012; Neuhaus et al., 2018) (α-SMA), platelet-derived growth factor receptor alpha (PDGFRα) (Monaghan et al., 2012; Gevaert et al., 2014) and the protooncogene c-Kit (Mccloskey and Gurney, 2002). It should be noted that c-Kit, the assumed specific marker for interstitial cells of Cajal (ICC), recently has been found to be expressed only on mast cells in urinary bladder (Gevaert et al., 2017). Upper lamina propria in the human bladder are characterized as Vim + /αSMA + /PDGFRα + /c-kit-. In contrast, DLP-ICs are Vim + /αSMA-/PDGFRα + /c-kit-. Detrusor-ICs have a similar phenotype to that of DLP-ICs (Monaghan et al., 2012; Gevaert et al., 2014; Steiner et al., 2018).

Considerable progress has been made regarding the cellular markers, calcium signaling, ion channels, and receptor expression of bladder ICs (Andersson and Mccloskey, 2014; Koh et al., 2018), but their exact physiologic functions in the bladder are not known. Based on morphology, spatial distribution, and limited functional data, ULP-ICs and DLP-ICs are thought to have important roles in afferent signaling processing in the bladder mucosa (Fry et al., 2007; Gevaert et al., 2011; Andersson and Mccloskey, 2014), whereas detrusor-ICs have been proposed to modulate detrusor spontaneous contractions or excitability (Koh et al., 2018).

In addition to their sensitivity to adenosine triphosphate (ATP), low pH, and acetylcholine (ACh) (Fry et al., 2007; Johnston et al., 2008), ULP-ICs have been shown to have mechanical sensitivity, and that mechanical stimuli such as stretch, shear stress, or hypotonicity, can evoke an increase in the intracellular Ca^2+^ concentration [(Ca^2+^)_*i*_] (Neuhaus et al., 2020). That study also suggested that ULP-ICs are the active elements in afferent signaling processing in the bladder. Active participation of ULP-ICs is also supported by a study that reported increased ATP release from cultured ICs from pig bladders by hypotonic stimulation (Cheng et al., 2011). In line with those findings, ULP-ICs network has been proposed to function as a stretch receptor for perception of physical and chemical stimuli (Wiseman et al., 2003; Vannucchi and Traini, 2018). Although purinergic (P2X3 or P2Y6) (Sui et al., 2006) or muscarinic receptors have been found in bladder ICs, it is not clear if other receptors that can perceive mechanical or chemical stimuli are present on LP-ICs.

Transient receptor potential (TRP) channels are important sensors for cells in response to mechanical and chemical stimuli or temperature change. TRPA1, TRPV1, TRPV2, TRPV4, and TRPM8 expressed in bladder sensory afferents or urothelial cells have pivotal roles in the sensory function of the bladder (Vanneste et al., 2021). However, few studies have investigated the expression and function of TRP channels in bladder ICs, particularly in ULP-ICs. Recently, the bladder ICs of humans, guinea pigs, and pigs have been shown express TRPA1 (Steiner et al., 2018). TRPA1 expression has also been found in Vim + ICs of the ureter (Weinhold et al., 2018) and prostate gland (Gratzke et al., 2010) of humans. However, whether TRPA1 is functionally active in LP-ICs is not known.

The recently recognized mechanically sensitive channels Piezo1/Piezo2 have been shown to be expressed in the human urothelium as well as in neurons of dorsal-root ganglia innervating the bladder, and have been implicated in mechanical sensory transduction in the bladder (Miyamoto et al., 2014; Marshall et al., 2020). We examined if these sensory channels (TRPA1, TRPV1, TRPV2, TRPV4, TRPM8, and Piezo1/Piezo2) are expressed in LP-ICs of the human bladder. Real-time reverse transcription-quantitative polymerase chain reaction (RT-qPCR), immunofluorescence staining, and Ca^2+^ imaging were utilized. Vim and α-SMA were used as molecular markers for ICs.

## Materials and Methods

### Ethical Approval of the Study Protocol

The study protocol was approved [KYLL-2016(GJ)A-0027] by the Ethics Committee of the Second Hospital, Cheeloo College of Medicine of Shandong University (Jinan, China). All patients provided written informed consent for their tissue to be used in our experiments.

Bladder tissues (body or dome) were obtained from 10 patients (four women, 6 men; mean age, 51.2 ± 10.1 years) undergoing cystectomy for bladder carcinoma. Bladder tissues were transported immediately to the laboratory for cell culture. Some of the tissues were fixed in formalin for immunofluorescence experiments.

### Culture of Lamina Propria-Interstitial Cells and Urothelial Cells

Culture of LP-ICs was conducted as described previously (Neuhaus et al., 2020). Briefly, after tumor-free bladder tissue (body or dome) was obtained, the bladder mucosa was dissected from the detrusor layer. Then, small fragments (∼1 mm^2^) were digested with trypsin in 37°C for 15 min, and digestion was stopped by 10% fetal bovine serum. Then, tissue was plated into tissue culture flasks for incubation in an atmosphere of 5% CO_2_ at 37°C. Smooth Muscle Cell Growth Medium 2 (Procell, Wuhan, China) was used as the culture medium to limit the growth of urothelial cells (Neuhaus et al., 2020). Cells from passage-2 to passage-8 were used. For Ca^2+^ imaging and immunocytochemistry experiments, cells were plated onto poly-L-lysine (Sigma–Aldrich)-coated glass coverslips (8 mm in diameter) and grown to 80% confluence and 50% confluence, respectively.

Culture of urothelial cells was undertaken as described in our previous study (Wen et al., 2021). Briefly, the mucosa (1.5 cm × 1.5 cm) dissected from the bladder wall was placed in Minimum Essential Medium containing dispase and HEPES (2.5 mg/mL) overnight at 4°C. Urothelial cells were scraped and placed in trypsin (0.25% *wt/vol*) for 5 min at 37°C, and dissociated by trituration. Cells were plated on poly-L-lysine-coated glass coverslips, and used for Ca^2+^ imaging 48–96 h after dissociation.

### Reverse Transcription-Quantitative Polymerase Chain Reaction and Real-Time Polymerase Chain Reaction

When the confluence of cultured cells reached >90% in culture flasks (25 mL), cells were treated with 0.25% trypsin and collected. Total RNA was extracted using the RNA Simple Total RNA kit (Tiangen, Beijing, China). The RNA concentration was determined using an ultraviolet spectrophotometer. Reverse transcription was conducted using a SPARKscript II RT plus Mix kit (Sparkjade, Qingdao, China) according to manufacturer instructions, and complimentary-DNA was amplified (40 cycles of denaturation for 15 s at 95°C, and primer annealing and elongation for 30 s at 60°C). RT-qPCR was carried out using a SYBR™ Green qPCR Mix (Sparkjade) and an QuantStudio™ 5 system (Thermo Fisher, Waltham, MA, United States). Specific primers for β-actin as well as TRP and Piezo channels were generated by BioSune (Shanghai, China) and the sequences of primers are shown in Table 1. Expression was measured using the 2^–ΔΔCt^ method.

**TABLE 1:** Oligonucleotide primer sets for quantitative real-time PCR (RT-PCR).

Name	Sequence (5′-3′)	Length	Tm
Piezo1 F	ACTTTCCCATCAGCACTCGG	20	64
Piezo1 R	CCACGAAGTCCTTGAGACCC	20	64
Piezo2 F	ACTGCTGGGAAAGTCGTTGT	20	60
Piezo2 R	TTGGGTGGAACTGCCTCTTG	20	60
TRPM8 F	AGCAGCGATGAAGACTTGGC	20	62
TRPM8 R	TGGGCGATGAAATGCTGGTC	20	62
TRPA1 F	CAGAAGACAAGTCCTGCCGA	20	62
TRPA1 R	TTGAGGGCTGTAAGCGGTTC	20	62
TRPV1 F	GAGAGACCTGTGCCGTTTCA	20	62
TRPV1 R	TCCCGTCTTCAATCAGCGTC	20	62
TRPV4 F	TCTCACCGCCTACTACCAGC	20	64
TRPV4 R	GTAGAGGGCTGCTGAGACGA	20	62
TRPV2 F	TCGCTGTATGACCTGGCTTC	20	62
TRPV2 R	GCTCCAAAACGACCATTCGG	20	62
β-Actin F	CATGTACGTTGCTATCCAGGC	21	57.6
β-Actin R	CTCCTTAATGTCACGCACGAT	21	55.6

*F: forward; R: reverse; Tm: melting temperature.*

For RT-PCR, Total RNA was extracted from the cultured cells using TRIzol (Invitrogen) and a DNA-free kit (Ambion). cDNA was synthesized using Superscript (Invitrogen). PCR was performed using Surestart *Taq* polymerase (Sparkjade).

### Immunofluorescence Staining

Sections of bladder tissue (5 μm) or cultured ICs on coverslips were fixed in 4% paraformaldehyde for 15 min following three times wash by PBS. Then blocked with 5% normal goat serum for 30 min and incubated with mixed two primary antibody (1:100; Table 2) at 4°C overnight on the shaker. Subsequently, washed with PBS and incubated with appropriate secondary antibody for additional 1 h at room temperature— Alexa Fluor 594-conjugated goat anti-mouse IgG (H + L; diluted 1:200 in phosphate-buffered saline; Elabscience Biotechnology, Wuhan, China) or fluorescein-conjugated goat anti-rabbit IgG (H + L; 1:50 dilution). To test the specificity of the primary antibodies, RNA interference for TRP/Piezo channels were applied. The siRNAs for TRP/piezo channels and the mismatch were produced by GenePharma (GenePharma, Shanghai, China), and their sequence were shown in Supplementary Table 1. They were transfected using transfection reagent siRNA-mate (GenePharma, Shanghai, China) according to the manufacturer’s protocol. Successful knock down of these channels was demonstrated by the qPCR experiments (mRNA level was decreased by 79.4% for Piezo1, 89.6% for Piezo2, 71.6% TRPM8, 79.4% for TRPA1, 75.8% for TRPV1, 73.2% for TRPV4 and 85.2% for TRPV2, respectively). The immunofluorescence for these channels were accordingly reduced (Supplementary Figure 1). Staining was analyzed using a confocal laser scanning microscope (Observer Z1; Carl Zeiss Microscopy, Baden Wurttemberg, Germany). Images were acquired using ZEN 2.1 (blue edition; Carl Zeiss Microscopy).

**TABLE 2:** Primary antibodies used in immunohistochemistry experiments.

Antibody	Host	Supplier	Code	Dilution
a-SMA	Rabbit	Abcam (Cambridge, United Kingdom)	ab124964	1:100
Piezo1	Rabbit	Affinity Biosciences LTD (JiangSu, China)	DF12083	1:100
Piezo2	Rabbit	Alomone Labs (Jerusalem, Israle)	APC-090	1:100
TRPA1	Rabbit	HUABIO (HangZhou, China)	ER1803-91	1:100
TRPM8	Rabbit	Novus Biologicals (Littleton, CO, United States)	NBP1-97311	1:100
TRPV1	Rabbit	Novus Biologicals (Littleton, CO, United States)	NB100-1617	1:100
TRPV2	Rabbit	Sigma-Aldrich (Munich, Germany)	SAB1101376	1:100
TRPV4	Rabbit	Novus Biologicals (Littleton, CO, United States)	NBP2-41262	1:100
Vim	Mouse	Invitrogen (Califonia, United States)	MA1-06908	1:100

### Ca^2+^ Imaging

Cultured IC on glass coverslips were loaded with Fura-2-acetoxymethyl ester (Fura 2-AM; 2 μM; Dojindo Laboratories, Tongren, Japan) for 30 min. Fura 2-AM was dissolved in Hank’s balanced salt solution containing (in mM): 138 NaCl, 5 KCl, 0.3 KH_2_PO_4_, 4 NaHCO_3_, 2 CaCl_2_, 1 MgCl_2_, 10 HEPES, and 5.6 glucose, pH 7.4. Ca^2+^ imaging was undertaken as described in our previous study (Wen et al., 2021). Briefly, coverslips were placed in a recording chamber. Fura 2-AM was excited with ultraviolet light alternately at 340 nm and 380 nm. Wavelength selection, timing of excitation, and image acquisition were controlled using MetaFluor^®^ (Molecular Devices, Sunnyvale, CA, United States). The ratio of the fluorescence signal measured at 340 nm divided by the fluorescence signal measured at 380 nm was used to measure the increase in [Ca^2+^]_*i*_. A significant increase in [Ca^2+^]_*i*_ was considered if the ratio change > 0.1.

### Adenosine Triphosphate Measurement

Samples of perfusate were collected 2 min before and immediately after agonist stimulation during calcium imaging study. The ATP concentration was measured using luciferin–luciferase bioluminescence, as described previously (Wen et al., 2021). Briefly, a mixture of 100 μL of luciferin–luciferase was added to 100 μL of sample according to manufacturer instructions using the CellTiterGlo™ Luminescent Cell Viability Assay kit (Promega, Fitchburg, WI, United States). Adenosine triphosphate detection was evaluated using the GloMax™ 20/20 luminometer (Promega).

### Single Cell Mechanical Stimulation

We referred Neuhaus et al. (2020) for single cell mechanical stimulation. Briefly, a motorized MP-285 Micromanipulator (Sutter Instruments, Novato, CA, United States) was used for controlling glass micropipette movement. Single cell was mechanically stimulated by deflection of the plasma membrane using a glass micropipette with a fine closed and rounded tip (about 2 μm). The micropipette was lowered in steps of 1 μm to induce the membrane deflection.

### Statistical Analyses

Data are the mean ± SEM. Significance was tested on raw data using a paired or unpaired *t*-test. Excel™ (Microsoft, Redmond, WA, United States) and Prism 8.0.2 (GraphPad, San Diego, CA, United States) were used for analyses. *P* < 0.05 was considered significant.

## Results

### Cultured Interstitial Cells Have the Phenotype of Vim+ αSMA+

**FIGURE 1:**
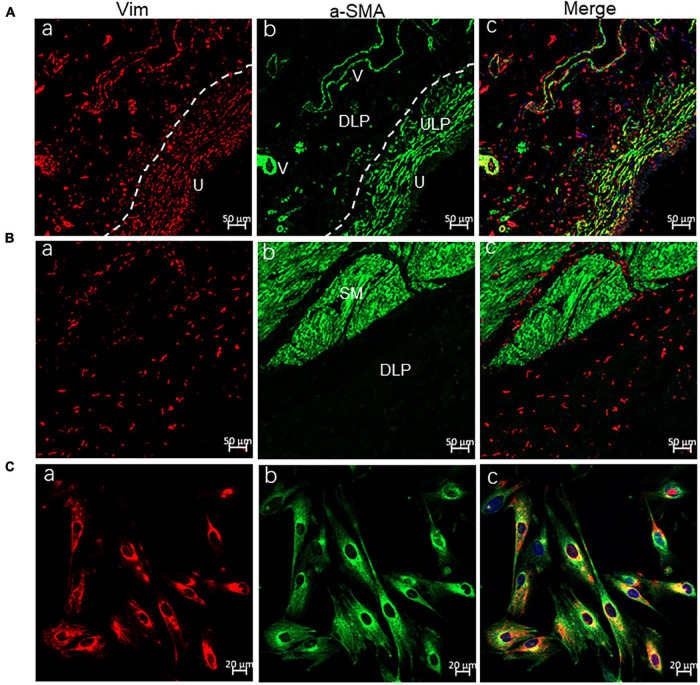
Confocal immunofluorescence for Vim (red) and α-SMA (green) showing ULP-ICs and cultured ICs having the phenotype of Vim+ α-SMA+. **(A,B)** Immunofluorescence of the bladder wall showing Vim+ (red) ICs distributed in the ULP [**(Aa)**, densely packed cells immediately beneath the urothelium], DLP [**(Ba)**, loosely distributed between the ULP and detrusor] as well within or between the detrusor muscle **(Ba)**. Vim+ staining is also seen in endothelial cells of blood vessels (V). α-SMA+ (green) staining is present on ULP-ICs **(Ab)** and the detrusor muscle [SM, **(Bb)**] but not on DLP-ICs or detrusor-ICs **(Bb)**. Perivascular smooth muscle also expresses α-SMA. Co-expression of Vim and α-SMA is shown in merged images **(Ac** and **Bc)**. **(Ca–Cc)** Immunofluorescence of Vim and α-SMA in cultured LP-ICs showing most of the Vim+ ICs are α-SMA+. DLP: deep lamina propria; ULP: upper lamina propria; U: urothelium; V: vessels; SM: smooth muscle. Dashed line indicates the transition between the ULP and DLP.

Most of our experiments were conducted on cultured ICs, so their identity was first examined using the commonly used IC markers Vim and α-SMA. Immuno-cytochemical imaging demonstrated that > 95% of cultured ICs were Vim+, and >90% Vim + ICs were α-SMA+ (Figures 1A–C). An immunohistochemistry-based study (Gevaert et al., 2014) on human bladder tissue revealed that α-SMA + Vim + ICs were located mainly in the ULP and packed densely in the sub-urothelial layer (Figure 1A). α-SMA-ICs were located at the DLP as well as between or within the detrusor (Figure 1B), which suggested that most of our cultured ICs were from the ULP. However, we cultured ICs from the bladder mucosa, so we could not exclude the presence of DLP-ICs. Thus, in the following sections, we describe them as “LP-ICs.”

In agreement with the results of a previous study (Neuhaus et al., 2020), ∼75% of cultured LP-ICs exhibited spontaneous Ca^2+^ activity (Supplementary Figure 2A). Usually, spontaneous Ca^2+^ activity was present ≥ 10 min after placement of cells in the perfusion chamber. ATP (100 μM) application could elicit a significant increase in [Ca^2+^]_*i*_ in these LP-IC (Supplementary Figure 2B).

### mRNA Expression of Transient Receptor Potential and Piezo1/Piezo2 Channels in Cultured Lamina Propria-Interstitial Cells

**FIGURE 2:**
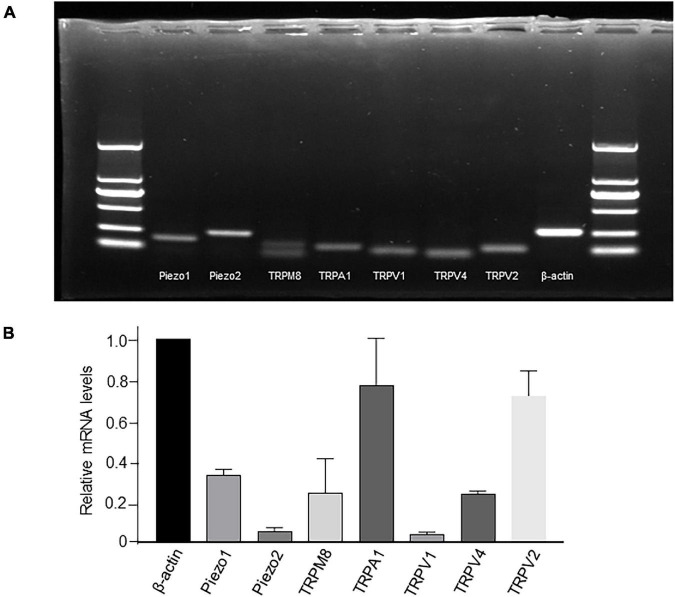
mRNA expression of TRP and Piezo1/Piezo2 channels in cultured LP-ICs. Total RNA was extracted from cultured LP-ICs and RT-PCR **(A)** or RT-qPCR **(B)** was conducted. **(A)** Typical images demonstrating that Piezo1, Piezo2, TRPM8, TRPA1, TRPV1, TRPV4 and TRPV2 channels are expressed in LP-ICs. **(B)** Relative mRNA expression of TRP and Piezo channels to that of beta-actin. Relative mRNA expression of TRPA1 and TRPV2 are the highest, and that of Piezo1, TRPV4 and TRPM8 are moderate. Summary data are the average from five experiments.

mRNA expression of TRP and Piezo channels was examined by simple PCR (Figure 2A) or RT-qPCR (Figure 2B). Among all the channels examined, the relative expression of TRPA1 and TRPV2 was the highest, Piezo1, TRPM8, and TRPV4 was moderate, whereas that of Piezo2 and TRPV1 was the lowest (Figure 2B).

### Cellular Expression of Transient Receptor Potential and Piezo1/Piezo2 Channels in Cultured Lamina Propria-Interstitial Cells

**FIGURE 3:**
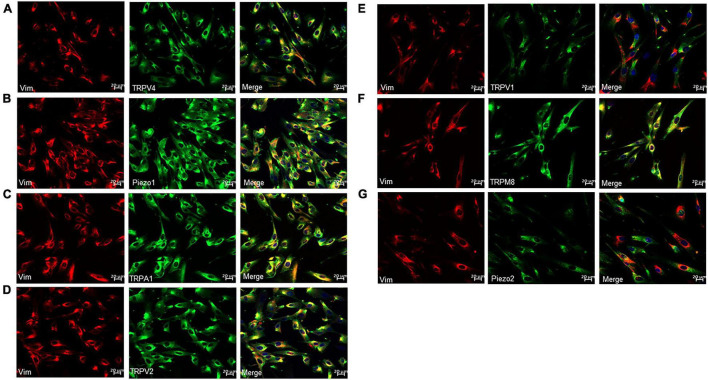
Immunofluorescence for Vim (red) and TRP or Piezo channels (green) in cultured LP-ICs. Double staining reveals the protein expression of TRPV4 **(A)**, piezo1 **(B)**, TRPA1 **(C)**, TRPV2 **(D)**, TRPV1 **(E)**, TRPM8 **(F)** and Piezo2 **(G)** in most of the Vim+ LP-ICs. The nucleus marker (DAPI) is stained in blue.

Protein expression of TRP and Piezo1/Piezo2 channels in cultured ICs was measured using immunocytochemistry. Prominent staining was observed for all examined channels in cultured ICs (Figures 3A–G).

### Functional Expression of Transient Receptor Potential and Piezo1/Piezo2 Channels in Cultured Lamina Propria-Interstitial Cells

**FIGURE 4:**
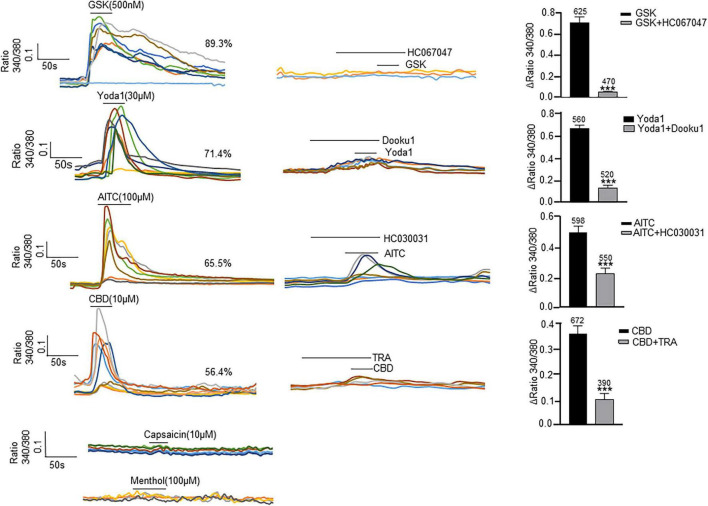
Agonists specific for TRP and Piezo1 channels elicit an [Ca^2+^]_i_ increase in cultured LP-ICs. Left columns are typical traces showing application of the TRPV4 agonist GSK1016790A (GSK, 500 nM), Piezo1 agonist Yoda 1 (30 μM), TRPA1 agonist AITC (100 μM) and TRPV2 agonist cannabidiol (CBD, 10 μM) to elicit a remarkable increase in [Ca^2+^]_i_, respectively. The TRPV1 agonist capsaicin (10 μM) and TRPM8 agonist methanol (100 μM) did not evoke a significant increase in [Ca^2+^]_i_. Agonists were applied for 30 s to 50 s. Middle columns are typical traces demonstrating that an increase in [Ca^2+^]_i_ induced by agonists of TRPV4, Piezo1, TRPA1 and TRPV2 was blocked by pretreatment with the corresponding antagonist. To avoid desensitization impacts, antagonists were applied in different coverslips with agonist experiments. Right columns are summary data for the blocking effects of antagonists. The number above each bar indicates the cell number. ***P < 0.001.

All the examined channels were highly permeable to Ca^2+^ (Vanneste et al., 2021), so their functional expression in cultured ICs was examined with Ca^2+^ imaging (Figure 4). Each agonist at its saturated concentration was applied before the presence of spontaneous Ca^2+^ activity. A specific agonist of the TRPV4 channel, GSK (500 nM), evoked a [Ca^2+^]_*i*_ increase in 89.3% of ICs (*n* = 700 cells from 13 coverslips), and this effect was blocked with pretreatment of HC-067047 (1 μM), a specific antagonist of TRPV4. Yoda 1 (30 μM), a specific agonist of Piezo1 channels, evoked a significant increase in [Ca^2+^]_*i*_ in 71.4% of ICs (*n* = 560 cells from 12 coverslips), and this effect was significantly blocked with pretreatment with the Piezo1-specific antagonist DooKu1 (10 μM). The TRPA1 agonist AITC (100 μM) evoked an increase in [Ca^2+^]_*i*_ in 65.5% of ICs (*n* = 598 cells from 15 coverslips), and this effect was blocked with pretreatment by the TRPA1-specific antagonist HC030031 (30 μM). A specific agonist of TRPV2, cannabidiol (10 μM), elicited an increase in [Ca^2+^]_*i*_ in 56.4% of ICs (*n* = 672 cells from 15 coverslips), and this effect was blocked significantly by pretreatment with Tranilast (10 μM), a TRPV2-specific antagonist. However, the TRPV1 agonist capsaicin (10 μM) and TRPM8 agonist methanol (100 μM) did not evoke a significant increase in [Ca^2+^]_*i*_. There is no commercially available agonist for Piezo2, so its functional expression could not be investigated.

Next, we compared the functional expression of the channels mentioned above in cultured ICs and cultured urothelial cells under identical recording conditions. Unexpectedly, only the TRPV4 agonist GSK (500 nM) evoked a significant increase in [Ca^2+^]_*i*_ in human urothelial cells, and responses were not found for agonists of the other channels (Supplementary Figure 3).

### Agonists for Transient Receptor Potential and Piezo1 Channels Evoked Adenosine Triphosphate Release From Lamina Propria-Interstitial Cells

**FIGURE 5:**
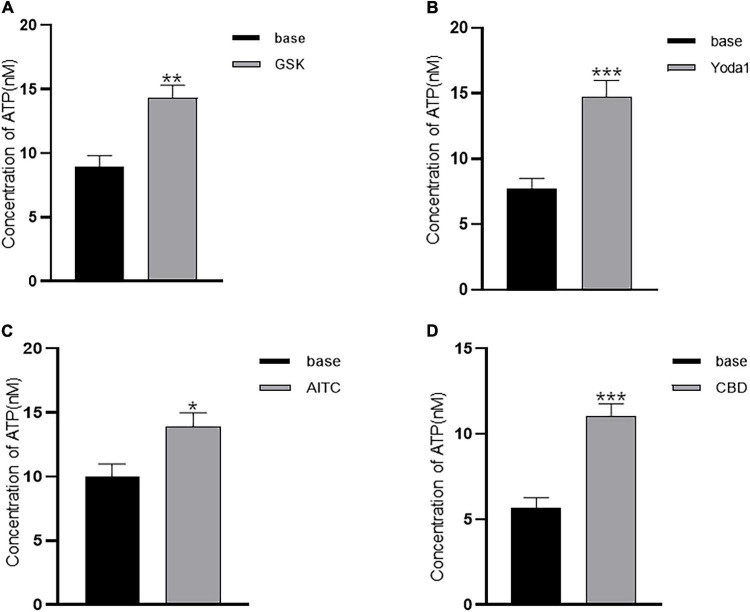
Agonists of TRP (A1, V2, and V4) and Piezo1 channels elicit ATP release from cultured LP-ICs. Samples of perfusate were collected 2 min before (base) and immediately after stimulation with GSK (500 nM) **(A)**, Yoda-1 (30 μM) **(B)**, AITC (100 μM) **(C)** and CBD (10 μM) **(D)**, and the ATP concentration in the perfusate was measured. The data for each figure is the average from 5 to 7 coverslips. *P < 0.05; **P < 0.01; ***p < 0.001.

Adenosine triphosphate (ATP) release in cultured ICs from pig bladders by hypotonic stimulation has been demonstrated (Cheng et al., 2011). We postulated that agonists of TRP and Piezo1 channels may elicit ATP release from LP-ICs. To test this possibility, the ATP concentration in the perfusate after agonist stimulation was measured. As expected, a significant increase in the ATP concentration was observed after application of GSK (500 nM), Yoda1 (30 μM), AITC (100 μM) and cannabidiol (10 μM) compared with that before application (Figures 5A–D). There was no significant change in the ATP concentration in coverslips without agonist stimulation, or after stimulation with capsaicin or methanol.

### Inhibition of TRPV2, TRPV4, Piezo1 Channels Reduced Stretch Induced Increase in [Ca^2+^]_i_

**FIGURE 6:**
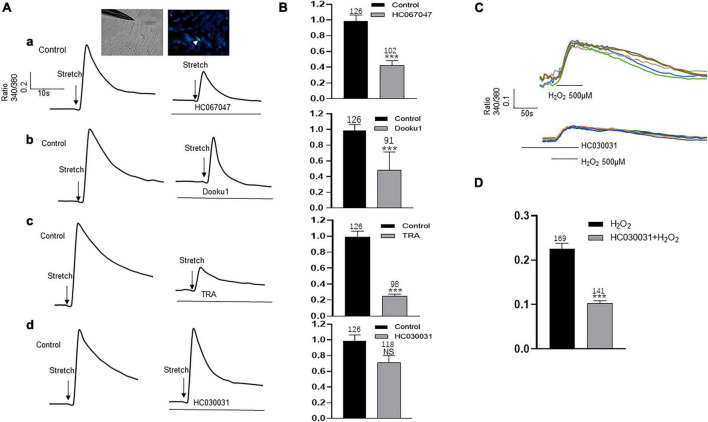
Antagonists of TRP (V2 and V4 but not A1) and Piezo1 channels inhibit stretch induced increase in [Ca^2+^]_i_ in cultured LP-ICs. **(A)**, Typical traces demonstrating that pretreatment with the antagonist of TRPV4 (**a**, HC067047, 1 μM), Piezo1(**b**, Dooku1, 10 μM), and TRPV2 (**c**, TRA, 10 μM) but not TRPA1 (**d**, HC030031, 30 μM) blocked stretch induced increase in [Ca^2+^]_i_. Inset indicates a cell was subjected to a step of 1 μm movement of a stimulation glass micropipette, and a fluorescence signal change was detected (indicated by a arrowhead). **(B)**, Summary data for the blocking effects of the antagonists. **(C)**, Typical traces showing that H_2_O_2_ (500 μM) induced an increase in [Ca^2+^]_i_, which was blocked by pretreatment with TRPA1 antagonist. **(D)**, Summary data for the blocking effects of TRPA1 antagonist. n above each bar indicates the cell number examined. ***p < 0.001.

Upper lamina propria (ULP-ICs) have been shown to have mechanical sensitivity, and mechanical stimuli such as stretch, shear stress, or hypotonicity, can evoke an increase in [Ca^2+^]_*i*_. In order to examine the involvement of above functional active TRPA1, TRPV2, TRPV4 and Piezo1 in mechanical responses of LP-ICs, the impacts of these channel antagonists on stretch (applied via a glass micropipette) induced [Ca^2+^]_*i*_ increase was investigated. TRPV2 antagonist (Tranilast, 10 μM), TRPV4 antagonist (HC-067047, 1 μM) and Piezo1 antagonist (DooKu1, 10 μM) reduced the stretch induced [Ca^2+^]_*i*_ increase by 75%, 57.7%, and 51.2%, respectively (Figures 6A,B). TRPA1 antagonist (HC030031, 30 μM) has no effect on stretch evoked response. Whereas it could reduce H_2_O_2_ (500 μM) induced [Ca^2+^]_*i*_ increase (Figures 6C,D).

## Discussion

We measured expression of TRPA1, TRPV1, TRPV2, TRPV4, TRPM8, and Piezo1/Piezo2 channels in human-bladder LP-ICs at mRNA, protein, and functional levels. To the author’s knowledge, this is the first study demonstrating the functional expression of TRPA1, TRPV2, TRPV4, and Piezo1 channels in human-bladder LP-ICs. Most importantly, activation of these channels resulted in ATP release from LP-ICs. Our observation suggests that LP-ICs could sense mechanical and chemical stimuli *via* these sensory channels, and then impact the activity of surrounding urothelial cells or nerve endings in a paracrine fashion. Our results provide further evidence for the active role of LP-ICs in the processing of sensory signals in the bladder mucosa.

In addition to ICs or stromal cells, bladder ICs have been called ICC, ICC-like, myofibroblast-like, fibroblast-like cells (Koh et al., 2018; Vannucchi and Traini, 2018), and telocytes (Vannucchi and Traini, 2018). This heterogeneity in terminology leads to considerable confusion between research teams working in this area. Nevertheless, ICs termed differently have a common property: positive staining with the broad mesenchymal marker Vim. Thus, Vim + cells were identified as ICs and the common term ICs was adopted in our study.

Two populations of ICs from the human bladder have been identified: α-SMA + /Vim + /PDGFRα + /TRPA1 + in the ULP, and α-SMA-/Vim + /PDGFRα + /TRPA1 + in the DLP and detrusor muscle (Monaghan et al., 2012; Gevaert et al., 2014; Steiner et al., 2018). Thus, α-SMA is the key marker to differentiate ULP-ICs from DLP-ICs. In agreement with this concept, αSMA + ICs were located mainly in the ULP of the human bladder wall (Figure 1B). For our cultured LP-ICs, >90% were α-SMA+, which suggests that cultured ICs were mainly from the ULP. The predominant population in cultured ICs was ULP-ICs, which could have been because DLP-ICs account for a minority of the LP-IC population in the human bladder (Figure 1B). α-SMA- cultured ICs (∼10% of the total) might be from the DLP.

For TRPV1 and TRPM8 expression, there is a disparity between the immunofluorescence staining (Figure 3) and the functional data (Figure 4). The lack of responses to capsaicin and menthol in LP-ICs (Figure 4) is in contrasts to the expression of TRPM8 and TRPV1 demonstrated by immunocytochemistry or RT-qPCR (Figure 2). Lamina propria may mimic urothelial cells that TRPV1 or TRPM8 expressions were demonstrated at mRNA level, but no functional expressions were found (Xu et al., 2009; Everaerts et al., 2010; Shabir et al., 2013). The reasons for the absence of capsaicin and menthol response in LP-ICs is not clear for us, probably because the mRNA expression level of TRPV1and TRPM8 are relatively low.

TRPA1-immunoactivity has been demonstrated in the bladder ICs of humans, guinea pigs, and pigs (Steiner et al., 2018). We demonstrated TRPA1 expression in human LP-ICs at mRNA and protein levels (Figures 2, 3). Furthermore, TRPA1 functional expression was found in 65.5% of LP-ICs in our study (Figure 4). Initially, TRPA1 was characterized as a noxious cold receptor. Subsequently, TRPA1 was identified as an important chemical sensor to painful or potentially harmful stimuli (Andersson, 2019). Thus, TRPA1 channels in LP-ICs may have an important role as sensors of toxic and irritant substances produced in bladder wall or pass from urine into the bladder wall if the urothelial barrier is disrupted during bladder infection or interstitial cystitis. In support of this idea, we found that H_2_O_2_, the product of oxidative stress (ROS), can induce an increase in [Ca^2+^]_*i*_ in LP-ICs, and this effect could be inhibited by pretreatment of TRPA1 antagonist (HC030031,30 μM; Figures 6C,D).

TRPV2, TRPV4, and Piezo1 are mechanical or stretch sensors (Andersson, 2019; Jiang et al., 2021). In our study, functional expression of these channels was found in most human LP-ICs. Most importantly, activation of these channels by their agonists promoted ATP release in LP-ICs. Studies have shown that ULP-ICs express the Cx43 protein and form gap junctions, thus behaving as a functional syncytium to propagate chemical or electrical signals (Fry and Vahabi, 2016). This ULP-IC functional network has also been proposed to act as a stretch receptor for the perception of local or bladder-wall distension (Wiseman et al., 2003; Vannucchi and Traini, 2018). In support of this notion, sub-urothelial ICs have been shown to have mechanical sensitivity, and that mechanical stimuli (e.g., stretch, shear stress, hypotonicity) induce an increase in [Ca^2+^]_*i*_ (Neuhaus et al., 2020). Our study further showed that stretch evoked intracellular Ca^2+^ increase could be inhibited by the antagonist of TRPV2, TRPV4, and Piezo1, respectively (Figure 6). This finding provides direct evidence that TRPV2, TRPV4, and Piezo1 may be the stretch sensors for ULP-ICs perceiving local or bladder filling-induced wall stretching.

Another important finding of our study is that activation of TRPA1, TRPV2, TRPV4, and Piezo1 channels by their agonists could elicit ATP release from LP-ICs. Bladder filling-induced ATP release from urothelial cells activating P2X3 receptors of sensory afferents has been considered the key underlying mechanism for generation of a bladder-filling sensation (Yu and de Groat, 2008). Given the close contact of ULP-ICs with sub-urothelial sensory nerves (Wiseman et al., 2003), we propose that ATP released from ULP-ICs may also have an important role in sensory afferent activation during bladder-filling. ULP-ICs are located immediately underneath urothelial cells, and urothelial cells and ICs are responsive to ATP (Supplementary Figure 2B). Thus, bidirectional communications between the urothelium and ULP-ICs may occur *via* ATP, and the two elements may form a stage for amplification of sensory signals and detection of bladder-filling (Fry et al., 2007).

In addition to the paracrine fashion (*via* ATP release) discussed above, the modulating effects of ULP-ICs on afferent activity in the bladder may also result from the mechanically contracting and stimulating impacts on sensory afferents. In the present study and previous studies (Monaghan et al., 2012; Gevaert et al., 2014; Steiner et al., 2018), ULP-ICs were found to contain the contracting element α-SMA. ULP-ICs will contract if [Ca^2+^]_*i*_ is increased in response to chemical or mechanical stimuli and, finally, alter gain of the sensory pathway.

Under identical recording conditions to that of LP-ICs, only TRPV4 was functionally expressed in our cultured human urothelial cells. This finding is consistent with other studies showing TRPV4 (but not TRPV1) being functionally expressed on human (Shabir et al., 2013), mouse (Everaerts et al., 2010) and guinea pig (Xu et al., 2009) urothelial cells. No functional expression of TRPV1 in human urothelial cells may suggest that capsaicin effects observed on human bladder may result from its action (activation or desensitization) on TRPV1 channels in primary sensory afferents (Fowler et al., 1992). However, expression of TRPV2 and Piezo1 channels in urothelial cells may have a species difference because functional expression of TRPV2 and Piezo1 are found in urothelial cells in mice (Everaerts et al., 2010) and rats (data not shown). Only TRPV4 is functionally expressed in human urothelial cells, which is in stark contrast to LP-ICs, in which TRPA1, TRPV2, TRPV4 and Piezo1 channels are functionally expressed. Given the important role of these channels in the sensing of chemical and mechanical stimuli, we propose that the role of the network of ULP-ICs may be even more important than that of urothelial cells in the perception of chemical or mechanical signals in the bladder mucosa.

In summary, we found that human suburothelial ICs functionally express TRPA1, TRPV2, TRPV4 and Piezo1 channels, and release ATP when these channels are activated. Our study suggests that LP-ICs can perceive stretch or chemical stimuli in the bladder LP *via* activation of TRPA1, TRPV2, TRPV4, and Piezo1 channels. Bidirectional communications may be present between LP-ICs and surrounding urothelial or sensory afferents in a paracrine manner. Given the recognized role of the urothelium in sensory function of the bladder, LP-ICs may work together with urothelial cells for perception and transduction of mechanical or chemical signals in human-bladder mucosa.

## Data Availability Statement

The raw data supporting the conclusions of this article will be made available by the authors, without undue reservation.

## Ethics Statement

The studies involving human participants were reviewed and approved [KYLL-2016(GJ)A-0027] by the Ethics Committee of the Second Hospital, Cheeloo College of Medicine of Shandong University (Jinan, China). All patients provided written informed consent for their tissue to be used in our experiments.

## Author Contributions

XZ and GC participated in the research design. MZ, ZC, LL, SZ, WS, and ND conducted the experiments. JW, JL, WW, SZ, WS, and NG performed the data analysis. XZ, GC, and MZ wrote the first manuscript, and all authors revised it critically to meet the standard for publication. All authors contributed to the article and approved the submitted version and are accountable for all aspects of this work.

## Conflict of Interest

The authors declare that the research was conducted in the absence of any commercial or financial relationships that could be construed as a potential conflict of interest.

## Publisher’s Note

All claims expressed in this article are solely those of the authors and do not necessarily represent those of their affiliated organizations, or those of the publisher, the editors and the reviewers. Any product that may be evaluated in this article, or claim that may be made by its manufacturer, is not guaranteed or endorsed by the publisher.

## Supplementary Material

The Supplementary Material for this article can be foundonline at: https://doi.org/10.6084/m9.figshare.15156495, https://doi.org/10.6084/m9.figshare.15156468, https://doi.org/10.6084/m9.figshare.15156501, and https://doi.org/10.6084/m9.figshare.15156498

Supplementary Figure 1 Immunofluorescence for TRP/Piezo channels weresignificantly reduced after knocking down their mRNA expressions **(A–G)**. Thecontrol images were from the sections treated with nontargeting siRNA.

Supplementary Figure 2 Cultured LP-ICs exhibit spontaneous Ca^2+^ activity and are responsive to ATP. **(A)** Typical traces showing 75% of ICs having spontaneous Ca^2+^ activity. **(B)** Typical traces showing ATP (100 μM) evoked a significant increase in [Ca^2+^]i in LP-ICs.

Supplementary Figure 3 Only a TRPV4 agonist evokes an increase in [Ca^2+^]i in cultured human urothelial cells. The duration and concentration for each agonist applied are identical to those applied for LP-ICs shown in Figure 4. The scale bar is applied to all figures.

Supplementary Table 1 The sequences of siRNAs for RNA interference.

## Abbreviations

AITC: Allyl isothiocyanate
ATP: adenosine triphosphate
DLP: deeper lamina propria
GSK: GSK1016790A
LP-ICs: Interstitial cells in the lamina propria
PDGFRα: platelet-derived growth factor receptor alpha
α-SMA: alpha-smooth muscle actin
TRP: Transient receptor potential
ULP: upper lamina propria.
